# Identification and validation of reference genes for qRT-PCR studies of the obligate aphid pathogenic fungus *Pandora neoaphidis* during different developmental stages

**DOI:** 10.1371/journal.pone.0179930

**Published:** 2017-07-03

**Authors:** Shutao Zhang, Chun Chen, Tingna Xie, Sudan Ye

**Affiliations:** 1 China Jiliang University, Zhejiang Provincial Key Laboratory of Biometrology and Inspection & Quarantine, Hangzhou, China; 2 Zhejiang Economic & Trade Polytechnic, Hangzhou, China; Institute for Sustainable Plant Protection, C.N.R., ITALY

## Abstract

The selection of stable reference genes is a critical step for the accurate quantification of gene expression. To identify and validate the reference genes in *Pandora neoaphidis*–an obligate aphid pathogenic fungus—the expression of 13classical candidate reference genes were evaluated by quantitative real-time reverse transcriptase polymerase chain reaction(qPCR) at four developmental stages (conidia, conidia with germ tubes, short hyphae and elongated hyphae). Four statistical algorithms, including geNorm, NormFinder, BestKeeper and Delta Ct method were used to rank putative reference genes according to their expression stability and indicate the best reference gene or combination of reference genes for accurate normalization. The analysis of comprehensive ranking revealed that *ACT1*and *18S*was the most stably expressed genes throughout the developmental stages. To further validate the suitability of the reference genes identified in this study, the expression of cell division control protein 25 (*CDC25*) and Chitinase 1(*CHI1*) genes were used to further confirm the validated candidate reference genes. Our study presented the first systematic study of reference gene(s) selection for *P*. *neoaphidis* study and provided guidelines to obtain more accurate qPCR results for future developmental efforts.

## Introduction

*Pandora neoaphidis* (Entomophthoromycotina, Entomophthorales) is an obligate aphid pathogenic fungus and has great potential for use in biological control applications[1–3]. Due to the ability to produce forcibly discharged infectious conidia, which germinate and penetrate the aphid through the cuticle, ultimately killing it, *P*. *neoaphidis* has a great value for the conservation and epizootioloical studies[2].

Although there are many sequences of *P*. *neoaphidis* deposited in the National Center for Biotechnology Information (NCBI) GenBank database, the vast majority of available genomic information is composed of partial gene and intron sequences developed for use in phylogenetic analyses[4]. Elucidation of the infection processes and pathogenicity by modern genomic tools is desirable because it provides clues for understanding the main elements of host-pathogen evolution and the function of virulence genes in pathogenic fungi[4,5]. However, the function of genes of interest (GOIs), especially gene expression of *P*. *neoaphidis* during the growth and infection process, is still lacking a detailed molecular understanding nowadays. Generally, a good understanding of the GOIs requires good knowledge of gene expression under a broad set of conditions. A diversity of genes can be up- or down-regulated during various developmental stages as well as pathogen-insect interactions[6]. Therefore, the study of *P*. *neoaphidis* gene expression in different conditions may provide useful information regarding the molecular mechanisms highlighting the epizootic ability enabling for the development of genetic engineering approaches and field application. Quantitative real-time reverse transcription polymerase chain reaction (qPCR) has been indicated to be a good method for measuring gene expression across different samples because of its advantages, e.g., high throughput capacity, sensitivity and specificity[7,8]. Furthermore, qPCR has been used for this purpose with several fungi species[9–12], even including *P*. *neoaphidis* which was described previously but using only one reference gene [5]. However, one constant challenge is the selection of suitable reference genes because it requires a species-specific solving process[13,14]. Moreover, it is not acceptable to use a single traditional gene nowadays, and instead the ‘gold standard’ is the normalization of gene expression levels by reference genes. Here, an ideal reference gene set should have constant expression across all the samples to be investigated, regardless of the developmental stage or any other biological or technical variability.

Additionally, the selection of inappropriate reference genes with variable expressions could lead to incorrect calculations of the expression of the target genes and, therefore, incorrect assumptions about the function of those target genes[15]. The identification of suitable reference genes is therefore essential for accurate transcript expression analysis. For this purpose, several statistical algorithms have been developed to identify the most suitable internal controls with the least expression variability; calculating their expression stability based on the qPCR data of candidate reference genes. These algorithms rank the putative reference genes, thereby to indicate the best reference gene or genes for normalization. The four most commonly used algorithms for assessing the appropriateness of reference genes are: geNorm[16], NormFinder[17], BestKeeper[18] and Delta Ct[19], and these algorithms have been widely used[20,21].

In this study, 13 putative housekeeping genes including*18S*(18S ribosomal RNA), *28S*(28S ribosomal RNA), *ACT1* (Actin-1), *ACTN1*(Actinin Alpha 1), *HPRT*(Hypoxanthine-guanine phosphoribosyltransferase), *EF1*(Elongation factor 1), *GAPDH*(Glyceraldehyde-3-phosphate dehydrogenase), *TBCE*(Tubulin-specific chaperone E), *LDHA*(L-lactate dehydrogenase A), *LSM1* (U6 snRNA-associated Sm-like protein LSm1), *HISTH4* (Histone H4), *ALG9*(Alpha-1,2-mannosyltransferase) and *DMA2* (E3 ubiquitin-protein ligase DMA2), were evaluated as potentially reliable reference genes in *P*. *neoaphidis* using qPCR. As fungal growth and infection of the host usually involve multiple developmental stages[22],the profiling of gene expression in multiple development stages is important for understanding the mechanisms of growth and pathogenesis. With this in mind, we tested four developmental stages, including conidia, conidia with germ tubes, short hyphae and elongated hyphae. The expression stability of each gene in all the samples was analysed by the geNorm, NormFinder, BestKeeper and Delta Ct programs, and the appropriate reference gene set for accurate normalization was then selected. Furthermore, for demonstrating the efficacy of the selected reference genes, we investigated the expression of cell division control protein 25 (*CDC25*), a key protein controlling entry into and progression through various phases of the cell cycle[23], and Chitinase 1(*CHI1*), responsible for the carbohydrate active enzymes[24]. The expression of these two genes were significantly different during different developmental stages, as it was already demonstrated in a previous RNA-seq experiment performed by our group (unpublished data).

## Materials and methods

### Isolate and culture conditions

*P*. *neoaphidis* was isolated from an infected green-peach aphid *Myzus persicae* (Sulzer) cadaver from HangZhou city, China, in 1998 using the ‘descending conidia’ method[25] and was designated as F98028[1]. It was subcultured on SEMA (Sabouraud dextrose agar [SDA, Oxoid, USA] supplemented with egg yolk and milk)[26]in 9cm diameter Petri dishes for 10d at 20°C in a 12:12 light: dark regime.

### *P*. *neoaphidis* sample preparations

Preparation of *P*. *neoaphidis* propagules at different developmental stages were modified slightly as previous description[5]. Primary conidia actively discharged from the mycelial mat during the period of peak sporulation were harvested into 0.01M sterile PBS(pH 7.3)[27]. After the conidia were centrifuged at 3170×g for 10 min, a concentration of 10^10^ conidia per ml was then inoculated into GLEN medium(0.4%glucose, 0.5% yeast extract, 0.65% lactalbumin hydrolysate,0.77% NaCl;)[28] in flasks at 20°C and 150 rpm in a light: dark (12:12) for different periods of time[1]. Samples were prepared at ~0h for conidia without morphological changes, after ~6h for conidia with germ tubes, after ~12h for early short hyphae (i.e., the length of the germ tube was no more than 200μm), and after 24h for elongated hyphae (i.e., the length of hyphae exceeded 200μm). Structures of fungal samples were observed microscopically to ensure that they were in the right developmental stage (S1 Fig, Appendix). Three biological replicates were performed for each developmental stage, i.e., 12 flasks in total were established.

### Total RNA isolation and cDNA synthesis

Total RNA was prepared and extracted mechanically for each sample using the RNeasy Mini Kit (QIAGEN, Cat. No 74104, Mississauga, Canada)according to the manufacturer’s instructions. The quality of the RNA was evaluated by 1% agarose gel electrophoresis to indicate the intact28S and 18S ribosomal RNA (rRNA) subunits without any contaminating genomic DNA. Nucleic acid concentrations were measured using a Nanodrop 1000 (Thermo Biotech, USA).

First-strand cDNA was synthesised using the PrimeScript^™^ RT reagent Kit with gDNA Eraser according to the manufacturer’s instructions (Takara, Cat.No.RR047A, China). Before the reverse transcription, RNase-free DNase I(Agilent Technologies, USA)was added immediately into the RNA samples in order to avoid potential errors from genomic DNA contamination. One μg total RNA was used for the cDNA synthesis. Subsequently, the cDNA was diluted into 20 ng μl^-1^with nuclease-free water(QIAGEN, Canada). The cDNA mixtures were diluted 1:10 with nuclease-free water and stored at -20°C for subsequent qPCR analysis.

### Primer design and validation

Nucleotide sequences of the *18S* and *28S* genes were downloaded from Genbank (http://www.ncbi.nlm.nih.gov/genbank/), while the other 11 candidate reference gene sequences, including *ACT1*, *ACTN1*, *HPRT*, *EF1*, *GAPDH*, *TBCE*, *LDHA*, *LSM1*, *HISTH4*, *ALG9*and *DMA2*,were acquired from *P*. *neoaphidis* RNA-seq dataset(unpublished data). All the Genbank accession numbers are shown in Table 1. Primer pairs for each gene were designed using Beacon Designer 7 software (Premier Biosoft, Palo Alto, CA,USA)(Table 1). The PCR parameters of each primer pair were evaluated as described in detail previously[5] for all the samples containing the same amount of cDNA from a cDNA mixture (mentioned above) as the template. Specificity of primer pairs for each candidate gene was confirmed using melting curve analysis and agarose gel electrophoresis.

**Table 1:** Primer and amplicon characteristics of RT-qPCR for the selected candidate reference genes and genes of interest(GOIs).

Gene	Accession No.	Primer sequence	Ampliconsize(bp)	R^2^	Primers efficiency
*18S*	HQ677591.1	F:CAAACCCGAGCAATAGTC R:GTAGGAGCCCGATAGTAAA	179	0.996	108.7%
*28S*	EF392405.1	F:TCTTATCAGTCTCAGCACCTTG R:TCTGTCAATCCTTACTATGTCTGG	181	0.996	104.0%
*ACT1*	KT715508	F:GACGAAGTTGCTGCTCTTG R:TCCCATACCAACCATGACAC	138	0.996	107.8%
*ALG9*	KT715507	F:TGAGGATATAAGCCCAAGAACG R:ATTGGGAACCTACCCATTTCG	96	0.998	107.7%
*ACTN1*	KT715516	F:GCGGAAATACAGCCTTAGC R:CTTCTTCCTGCCGTTCTATC	178	0.998	98.2%
*EF1*	KT715517	F:CCGATGTCTATGTCTGCTAAGG R:TTGGCGAGGCTCAAATCATG	193	0.999	98.7%
*GAPDH*	KT715509	F:GGTAGTTATCTCTGCTCCTTCTG R:CAGTGGTAGCGTGACAAGTAG	194	0.996	101.4%
*HISTH4*	KT71551	F:TGCTATTCGTCGTCTCGC R:GCATCACGGACAACTGACTCTA	111	0.998	109.7%
*HPRT*	KT715515	F:AGGTGGGCTCATAACTTTGG R:GGTCCTCTAGTTGTATGTTGTG	295	0.998	100.7%
*LDHA*	KT715510	F:CCTGGTTGGTCGTAATCG R:CGGAGTCTCTGAGTATCAAG	178	0.996	100.3%
*TBCE*	KT715514	F:TGGTAAGCGTCAGCGATACAATGG R:CCGTCTAGCCGTAGCGTATTAAGTG	172	0.997	100.6%
*LSM1*	KT715513	F:GGGTGAAGTGGACGAAGAAATC R:CCTTGAGAGTGAAGAACATGGTG	141	0.998	108.0%
*DMA2*	KT715511	F:GCGTTCGGCATTCTTGATG R:CCTGTTGTCGTTCCTGTTTC	157	0.996	103.1%
*CDC25* *CHI1*	KX197113 KX197114	F:CCAAGTCCCTTACAACCTTATCG R:CCACAGTGTCATCGGATTGAG F:GCCTGCTTGR:GTCTGTATGG R:GTAGAAGGAGGTTCGCTTGG	185 282	0.997 0.997	101.2% 104.5%

### Quantitative PCR

The qPCR was performed on an iQ^™^5 multicolour real-time PCR detection system (Bio-RAD, USA) using MicroAmp^™^ Reaction Tubes sealed with MicroAmp Optical 8-cap Strip(ABI, USA). The reaction mix contained a 20 ng template of cDNA, SYBR Premix Ex Taq^™^II(12.5 μl; Takara, China) and 400 nM each of the forward and reverse primers (Sangon, China)in a final volume of 25 μl. The PCR was accomplished after a 10 min activation/denaturation step at 95°C, followed by 40 cycles of 30 s each at 95°C, 60 s each at 60°C, and 30 s each at 72°C. Fluorescence was detected at each polymerisation step. A cDNA mixture template containing equal amounts of cDNA from all samples was used to evaluated the PCR efficiency of each primer pair. The cDNA mixture template (from 10 ng to 1 pg) was diluted as ten-fold series. After the PCR amplification, a melting curve analysis was performed to check the amplification specificity. Three technical replicates were performed for each sample. Two control samples, the no-template control (NTC), and the *P*. *neoaphidis* genomic DNA were included with each primer pair tested.

### Statistical analyses

The expression levels of the candidate reference genes were determined from the Cq values (quantification cycle value), i.e., the number of PCR cycles at which the quantity of amplified targets reached a specific threshold level of detection. The expression stability of the reference genes was evaluated by analysing all the raw Cq values using the four programs: geNorm[16], NormFinder[17], BestKeeper[18] and the comparative Delta Ct[19]. geNorm can automatically calculate the gene expression stability measure *M* which is the mean pair-wise variation for a given gene compared with all other tested genes. Gene with high *M* values has greater variation in expression, and the rank of tested genes is evaluated via a stepwise exclusion process. NormFinder uses a model-based variation of a gene as its stability value and can rank a set of genes according to their stability values. BestKeeper algorithm evaluates the expression stability of a candidate reference gene by repeated pair-wise correlation analysis of the gene relative to all other candidate reference genes. Further software Delta Ct: a method similar to that described by Vandesompele et al [16], whereby compared ‘pairs of genes’ for evaluating the expression stability. Besides, in order to balance the ranking of the candidate reference genes across the different algorithms, the expression stability was evaluated with the RefFinder tool(http://www.leonxie.com/referencegene.php?type=reference) to determine the overall comprehensive stability value for each candidate reference gene[29]. Additionally, the optimal number of genes needed for accurate normalization—namely the normalization factors NF_n_, which is the geometric mean of the n most stable genes—was measured stepwise with the geNorm program[16]. Pairwise variations (V_n/n+1_) between two subsequent normalization factors (NF_n_/NF_n+1_) were calculated to indicate the effect of adding the (n+1)^th^ gene for normalization. Here, if V_n/n+1_ is larger than the recommended threshold of 0.15, that means that adding the (n+1)^th^ gene has a significant effect and thus this target gene is considered as a reliable reference gene.

### Validation of reference genes

In order to validate there liability of the selected reference genes as determined by RefFinder, the relative expression profiles of *CDC25*, a cell division control protein 25involved in controlling entry into and progression through various phases of the cell cycle, and *CHI1*, a gene of the carbohydrate active enzymes, were measured and normalized with the most stable and least stable reference genes. The RT-qPCR amplification conditions were the same as described above and primer pairs were shown in Table 1. The relative expression data was calculated according to the 2^−ΔΔCq^ method and presented as fold change [30]. Samples from conidia stage were used as reference samples. In case of statistical significance, the relative quantitation (RQ)results were compared among the more stable and more unstable reference genes with results of RNA-seq analysis by Mann–Whitney U test. Statistical analyses were performed using the computer program DPS System [31].

## Results

### Quantification and expression levels of the candidate reference genes

*18S*, *28S* and 11 housekeeping genes pre-selected from the RNA-seq database, including *ACT1*, *ACTN1*, *HPRT*, *EF1*, *GAPDH*, *TBCE*, *LDHA*, *LSM1*, *HISTH4*, *ALG9*and *DMA2*, were tested as references for the gene expression studies. There was a single peak in the melting curve of each qPCR reaction, but there were no signals detected within the controls, indicating that primer dimers or other non-specific amplification products had not appeared(S2A Fig, Appendix). The sizes of the amplicon products generated from specific primers were in the range 96–295 base pairs(Table 1). R^2^ presented a good linear relationship between the Cq values and the relevant relative amount of cDNA in all the qPCR reactions. Correspondingly, the amplification efficiencies were estimated to be 98.2–109.7%(Table 1). The specificity of the primer pairs for each candidate reference gene was identified using agarose gel electrophoresis(S2B Fig, Appendix). Thus, the 13primers were shown to be specific and appropriate for qPCR analysis.

**Fig 1:**
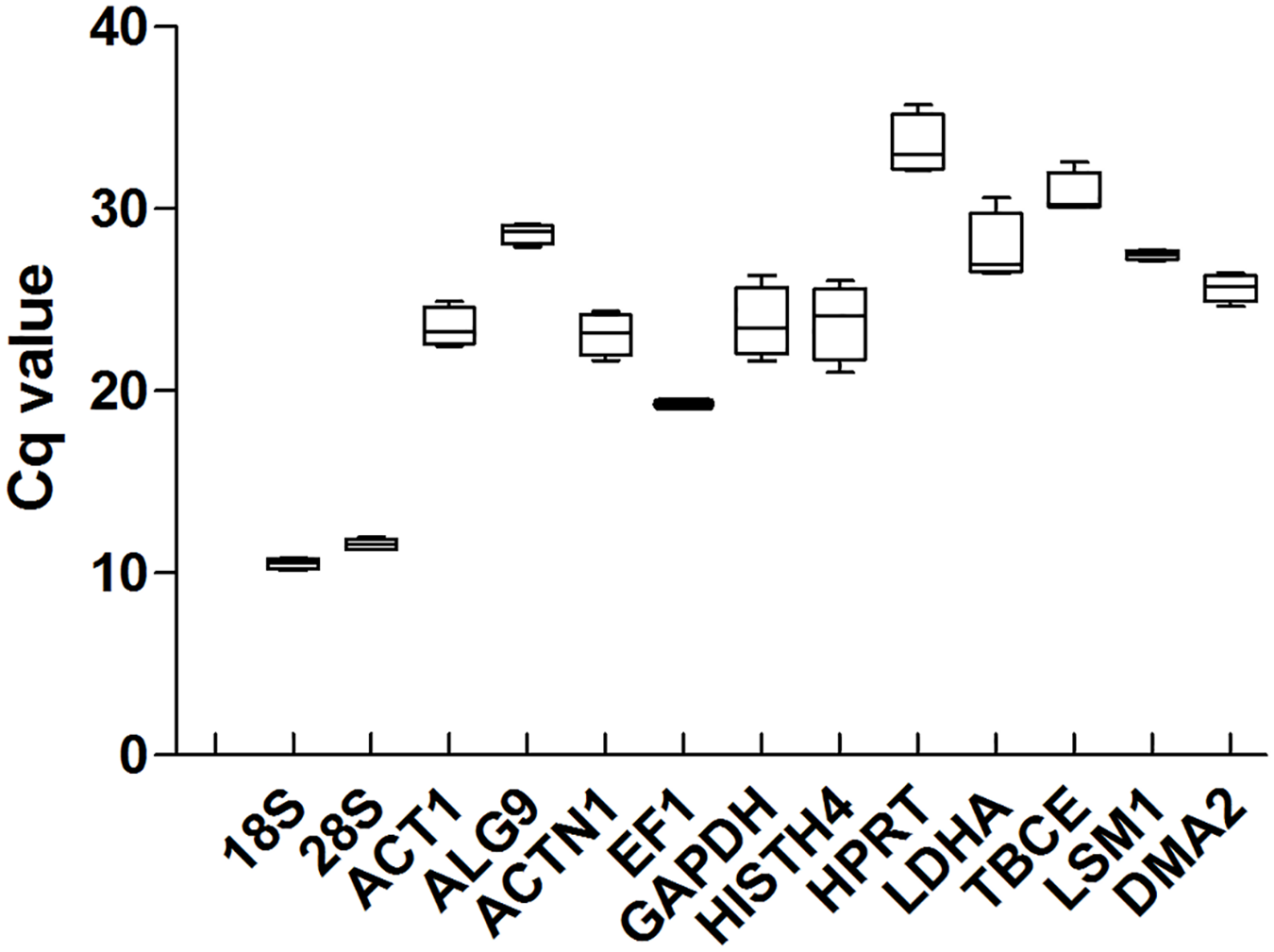
Distribution of the raw Cq (quantification cycle)values of the 13 candidate reference genes at four developmental stages. The lower and upper boundaries of the box (interquartile) represent the 25% and 75% percentiles, respectively. Whiskers indicate the maximum and minimum values. The median is depicted by the line across the box.

The calculated expression levels (Cq values) of the 13 genes evaluated varied from 10.16 to 35.73, which represents quite a large degree of variation (Fig 1). *18S*, *28S*, *EF1*and *LSM1*showed a compact distribution of Cq values, displaying a relatively low variation among the four developmental stages. Some of these, i.e., *GAPDH*, *HISTH4*and *HPRT*, as well as the *LDHA* gene showed a slightly higher dispersion of their Cq values, thus displaying a relatively high variation among the different developmental stages.

### Candidate gene expression stability during four developmental stages

**Fig 2:**
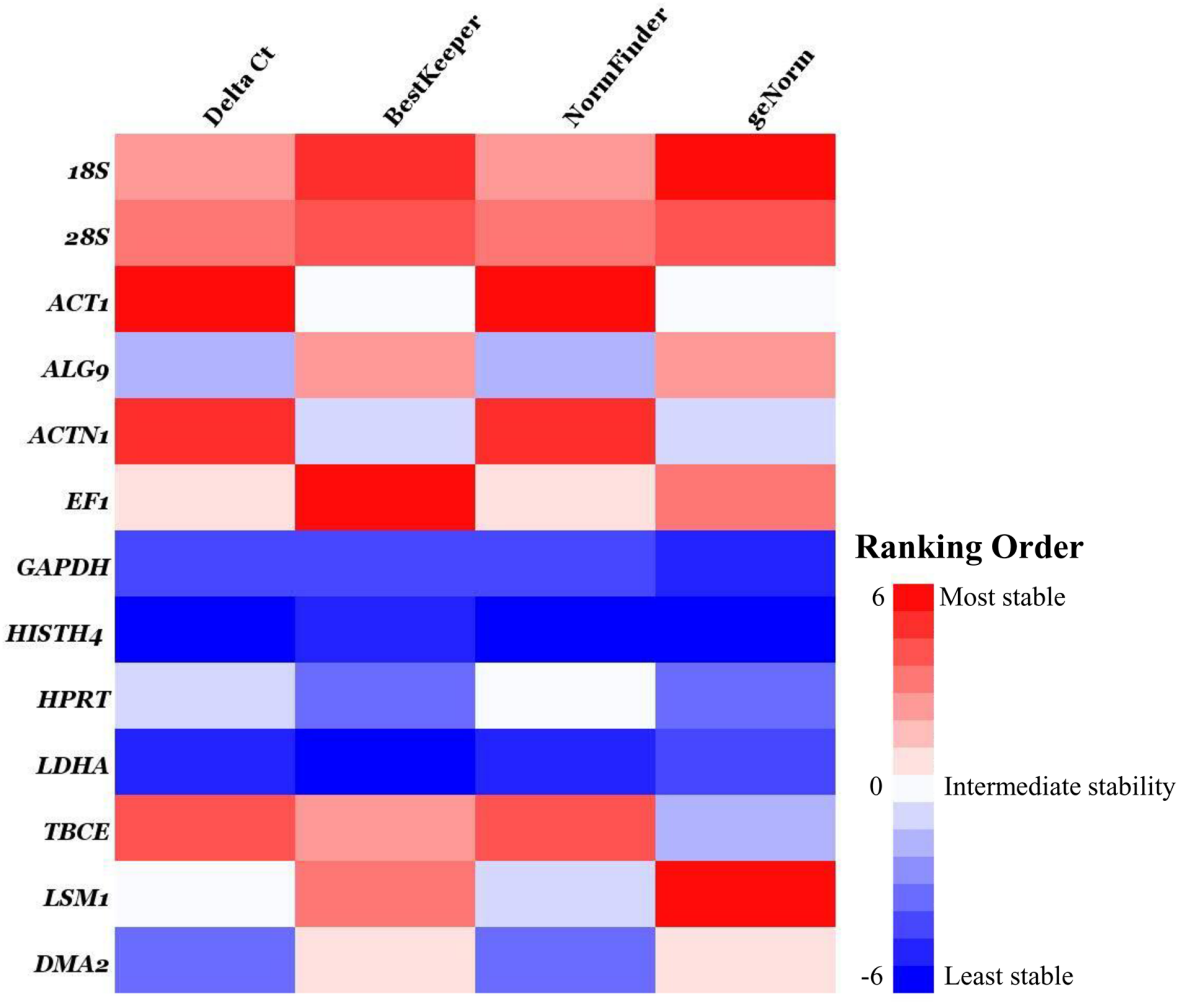
Heat map of the ranking in expression stability calculated by geNorm, NormFinder, BestKeeper and Delta Ct. The analysis was performed separately for each 9 sample group under the conditions examined. The 13 candidate references are coloured based on their stability in different saturations. Red represents the most stable and blue the least stable in ranking order.

Using the algorithms geNorm, NormFinder, BestKeeper and Delta Ct,the results of the stability tests were different(Fig 2). The heat map showing the ranking order indicates the most to the least stable, ranging from red to blue. The stability values calculated by geNorm, NormFinder, BestKeeper and Delta Ct are listed in S1 Table, respectively. The NormFinder and Delta Ct algorithms led to similar classifications,ranking*ACT1*,*ACTN1*and *TBCE* as the three most stable genes. However, the ranking order of stability calculated by the BestKeeper algorithms was:*EF1*, *18S*,*28S*, *LSM1*, *ALG9*,*DMA2*, *ACT1*, *ACTN1*, *TBCE*, *HPRT*, *GAPDH*, *HISTH4*, *LDHA*. Additionally, the ranking order of stability calculated by the geNorm algorithms was:*18S*,*LSM1*, *28S*, *EF1*, *ALG9*,*DMA2*, *ACT1*, *ACTN1*, *TBCE*, *HPRT*, *LDHA*,*GAPDH*, *HISTH4*. Unexpectedly, as calculated by geNorm, the *M* values of these 13 genes were below the recommended cut-off value of 1.5, even for the *GAPDH* gene, but also for *LDHA* and *HISTH4*, both of which ranked among the least stable genes, consistent with the behaviour calculated by the other three algorithms.

**Fig 3:**
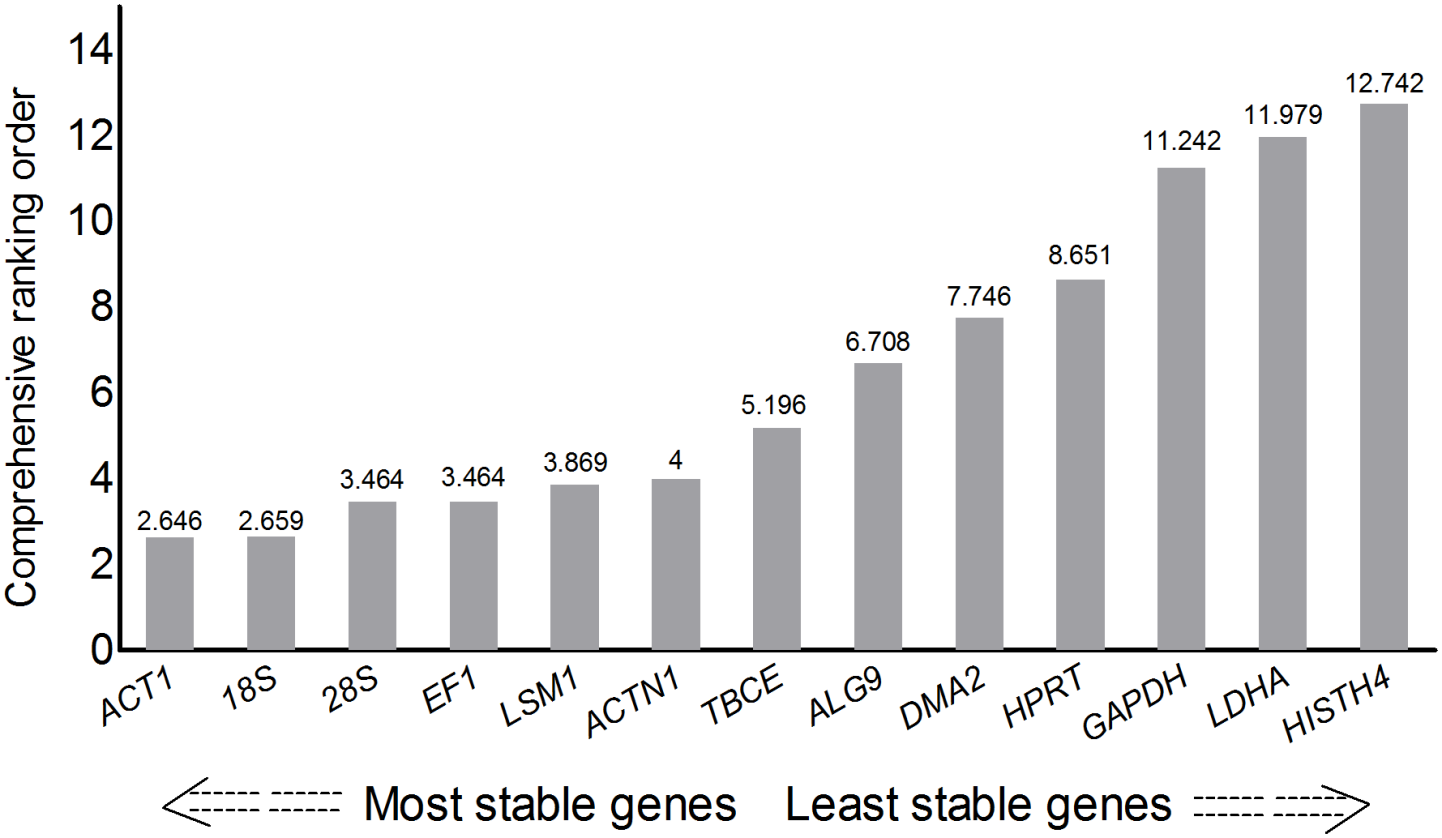
Comprehensive ranking of the candidate reference genes. The expression stability was evaluated with the RefFinder tool(http://www.leonxie.com/referencegene.php?type=reference) to determine the overall comprehensive ranking order for each candidate reference gene.

To reinforce this result, RefFinder, a web-based tool that integrates four algorithms: geNorm, NormFinder, BestKeeper and the comparative Delta Ct method—was also employed to balance the ranking of the candidate genes. As can be seen in Fig 3, the overall stability was calculated as the geometric mean of their weights. The ranking order from the most to least stable was:*ACT1*, *18S*,*28S*, *EF1*, *LSM1*, *ACTN1*,*TBCE*, *ALG9*,*DMA2*, *HPRT*, *GAPDH*, *LDHA*, *HISTH4*.

### Determination of the optimal number of reference genes

**Fig 4:**
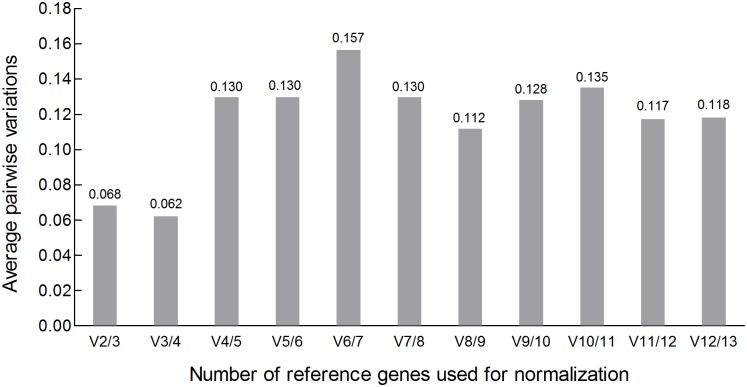
Optimal number of reference genes in different developmental stages of *P*. *neoaphidis* for normalization as evaluated by geNorm. Pairwise variations (V_n/n+1_) between two subsequent normalization factors NF_n_(geometric mean of the expression levels of n reference genes) and NF_n+1_ in the course of a stepwise inclusion of reference genes were calculated to indicate the effect of adding the (n+1)^th^ gene for normalization.

The optimal number of reference genes was evaluated as required for accurate normalization in the four developmental stages using the geNorm program. As shown in Fig 4,a combination of the three most stable genes,*ACT1*,*18S* and *28S*, has the lowest pairwise variation value (V_3/4_) of 0.062, which is lower than the threshold of 0.15. If *28S*is excluded, V_2/3_ = 0.068 is higher than V_3/4_,but is still lower than the threshold of 0.15. Therefore, a set with two reference genes (*ACT1* and *18S*) is sufficient for accurate normalization of the transcript levels in all of the samples.

### Validation of reference genes

**Fig 5:**
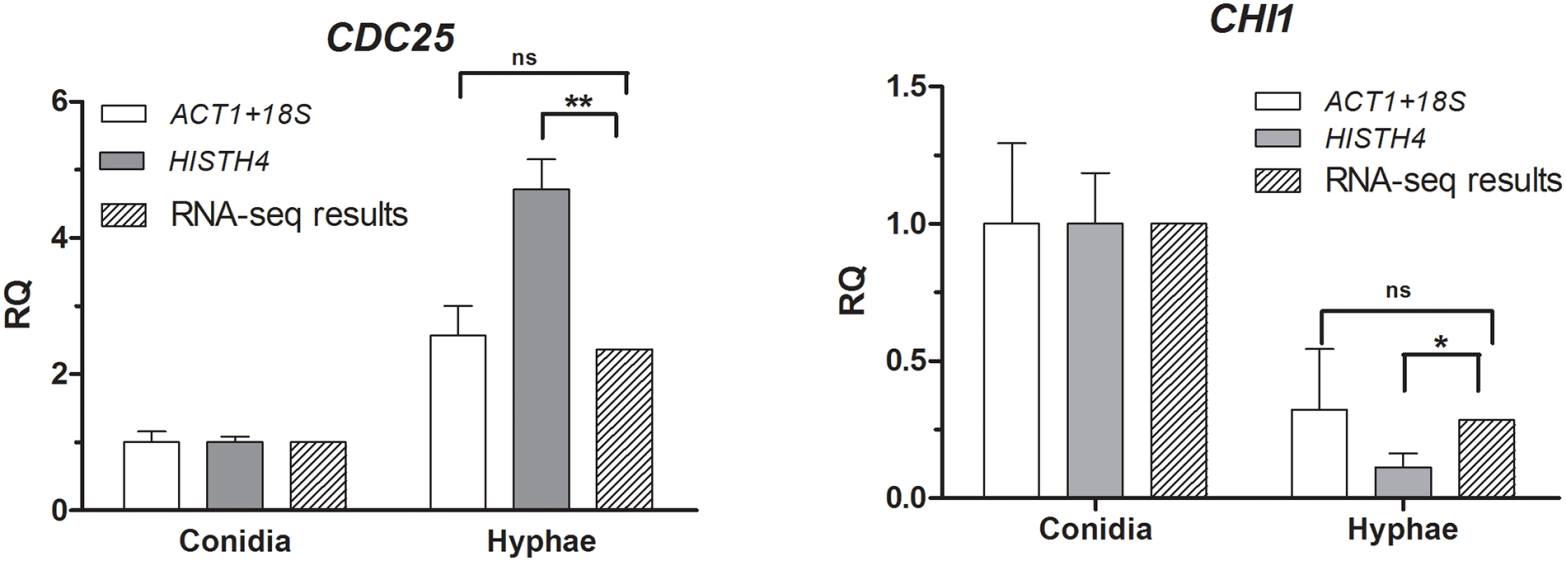
Expression profile of *CDC25* and *CHI1*in *P*. *neoaphidis* under conidia stage and elongated hyphae stage. The best stable combination of reference genes (*ACT1* and *18S*, white bars) and the least stable reference gene (*HISTH4*,black bars) were used to normalize the expression data. The relative quantification (RQ) of *CDC25* and *CHI1*(conidia stage VS elongated hyphae stage) obtained from RNA-seq analysis were marked as grey bars and chosen as standard for identifying their stability. Samples from the conidia stage were used as reference samples. The data show the mean expression ± standard deviation calculated from three biological replicates.

The quantification was realized using normalization by the combination of the two most stable genes as determined above and one of the least stable genes in developmental stages, i.e.,“*ACT1*+*18S*” and *HISTH4*, respectively. As shown in Fig 5, we observed that *CDC25*gene was indeed up-regulated and *CHI1* gene was down-regulated regardless using the set of “*ACT1*+*18S*” reference genes or *HISTH4*reference gene to normalize. The relative expression data of *CDC25* and *CHI1* were calculated according to the 2^−ΔΔCq^ method based on the qPCR data and presented as fold change. Furthermore, the relative quantification (RQ) results obtained for *CDC25* and *CHI1* using the “*ACT1*+*18S*” gene set for the normalization were not significantly different from that obtained from our RNA-seq database (u = 0.72, p>0.05; u = -0.37, p>0.05, respectively). In contrast, using *HISTH4* for expression normalization gave rise to very different results compared to that from RNA-seq (u = 8.23, p<0.01; u = 2.38, p<0.05, respectively). These results confirm the suitability of the “*ACT1*+*18S*” gene set for normalization purposes.

## Discussion

Fungal growth and infection of the host usually involves multiple developmental stages[22]. In this study, we evaluated the profiling of gene expression in four development stages for understanding the mechanisms of growth and pathogenesis. Using RNA-seq databases of *P*. *neoaphidis*, we carried out a pre-selection of the stable genes with a fold change(FC) equal to one between hyphae RNA-seq database and conidia RNA-seq database. Thirteen classical housekeeping genes were used as candidate reference genes for evaluating stability studies in *P*. *neoaphidis* (Table 1).

The selection of stable reference genes is a critical influence on the accuracy of qPCR[32]. Through the stability analyses, we identified that the *ACT1* and *18S*reference genes were the most suitable for normalization of the qPCR data from the multiple developmental stages. Those encoding rRNA genes are most conserved and constitute about 85–90% of the total cellular RNA in eukaryotes[33]. The *18S* gene—a classic housekeeping gene—has been widely used as a reference gene in the analysis of gene expression, e.g., in the entomopathogenic fungus *Beauveria bassiana*[9,34], *P*. *neoaphidis*[5]and other species[35,36]. Additionally, the *ACT1* gene encodes the single essential gene for actin, which is a ubiquitous, conserved cytoskeletal element critical for many cellular processes[37]. Although *ACT1* was not always encourage to use as reference gene since stationary phase samples showed a more significant drop of *ACT1* mRNA[38], they are still assumed to be constitutively expressed and widely used as reference genes[39,40]. In this study, *ACT1* is appropriate reference gene for evaluating expression stability during four developmental stages in *P*. *neoaphidis*.

Furthermore, by using simply a single algorithm to analyse the biological stability of expression in candidate reference genes, we often have difficult to evaluate and chose the best reference gene exhaustively [41]. Therefore, in this study, we adopted a comprehensive online tool based on four algorithms involved geNorm, NormFinder, BestKeeper and Delta Ct. Although the stability of the 13 candidate reference genes differed among the four software programs, reliable reference genes were identified for use in gene expression analyses. The overall ranking order—from the most to least stable—was *ACT1*, *18S*,*28S*,*EF1*, *LSM1*, *ACTN1*,*TBCE*, *ALG9*,*DMA2*, *HPRT*, *GAPDH*, *LDHA*, *HISTH4*, as highlighted by the comprehensive RefFinder tool. These algorithms did not always return identical results, especially for geNorm and BestKeeper compared to the NormFinder and Delta Ct methods. In addition, we obtained an unexpectedly identical stability of the candidate reference genes in different culture nutrition, indicating that they are all suitable for normalization (unpublished data). This may also be the reason for the different algorithms giving different normalization results. Furthermore, to evaluate the profiling of gene expression in growth period and pathogenesis stage, the relative quantitation of two relative genes including *CDC25* and *CHI1*, were measured using the set of “*ACT1*+*18S*” reference genesand*HISTH4*reference gene under conidia stage and hyphae stage. Compared with the RNA-seq database, results showed that the use of unsuitable references leads to differences in the relative expression profile. These results further confirmed the importance of validating reference genes prior to experimental applications.

## Conclusions

In this study, our results suggest that the *ACT1* and *18S* gene set would be the most reliable for normalizing the expression levels in multiple stages of the entomophthoralean fungus *P*. *neoaphidis* with the accurate and widespread method: qPCR. However, a further complication is present in that fungus-insect interactions involved fungal propagules and the host. Thus, further studies are required to assess and validate the identified reference genes suitable for the expression of GOIs during fungus-insect interactions.

## Supporting information

S1 Table Stability values calculated by geNorm, NormFinder, BestKeeper and Delta Ct programs. (DOCX)

S1 Fig Image of *P*. *neoaphidis* propagules. (DOCX)

S2 Fig Confirmation of primer specificity and amplicon length for thirteen candidate reference genes. (DOCX)
