# Supplementary material for: Identification and validation of reference genes for qRT-PCR studies of the obligate aphid pathogenic fungus Pandora neoaphidis during different developmental stages
Source: PLoS One. 2017 Jul 3;12(7):e0179930. doi: 10.1371/journal.pone.0179930 (PMC5495205; doi:10.1371/journal.pone.0179930)
Supplement: S1 Table — (DOCX) [file pone.0179930.s001.docx]

**Supporting Information**

**S1 Table.** Stability values calculated by geNorm, NormFinder, BestKeeper and Delta Ct programs.

| **Delta Ct** | | **BestKeeper** | | **NormFinder** | | **geNorm** | |
| --- | --- | --- | --- | --- | --- | --- | --- |
| Genes | Average of STDEV | Genes | std dev [+/- CP] | Gene | Stability value | Gene | Stability value |
| *ACT1* | 0.96 | *18S* | 0.21 | *ACT1* | 0.195 | *18S* | 0.209 |
| *ACTN1* | 1.04 | *28S* | 0.21 | *ACTN1* | 0.416 | *LSM1* | 0.209 |
| *TBCE* | 1.07 | *ACT1* | 0.72 | *TBCE* | 0.509 | *28S* | 0.223 |
| *28S* | 1.1 | *ALG9* | 0.37 | *28S* | 0.815 | *EF1* | 0.251 |
| *18S* | 1.11 | *ACTN1* | 0.83 | *18S* | 0.816 | *ALG9* | 0.414 |
| *EF1* | 1.11 | *EF1* | 0.05 | *EF1* | 0.861 | *DMA2* | 0.538 |
| *LSM1* | 1.19 | *GAPDH* | 1.31 | *HPRT* | 0.866 | *ACT1* | 0.716 |
| *HPRT* | 1.2 | *HISTH4* | 1.41 | *LSM1* | 0.999 | *ACTN1* | 0.815 |
| *ALG9* | 1.25 | *HPRT* | 1.16 | *ALG9* | 1.034 | *TBCE* | 0.885 |
| *DMA2* | 1.38 | *LDHA* | 1.43 | *DMA2* | 1.185 | *HPRT* | 0.978 |
| *GAPDH* | 1.44 | *TBCE* | 0.89 | *GAPDH* | 1.255 | *LDHA* | 1.082 |
| *LDHA* | 1.49 | *LSM1* | 0.22 | *LDHA* | 1.283 | *GAPDH* | 1.154 |
| *HISTH4* | 1.67 | *DMA2* | 0.5 | *HISTH4* | 1.513 | *HISTH4* | 1.233 |
