# Supplementary material for: Identification and validation of reference genes for qRT-PCR studies of the obligate aphid pathogenic fungus Pandora neoaphidis during different developmental stages
Source: PLoS One. 2017 Jul 3;12(7):e0179930. doi: 10.1371/journal.pone.0179930 (PMC5495205; doi:10.1371/journal.pone.0179930)

**Supporting Information**

**S1 Fig**. Image of *P. neoaphidis* propagules. To prepare propagules at different developmental stages, primary conidia produced by a mycelial mat were incubated in GLEN broth. The cultures were centrifuged to harvest conidia at 0 h (A), conidia with germ tubes at 6 h (B), early hyphae at 12 h (C) and elongated hyphae at 24 h (D). Scale bar: 50µm.


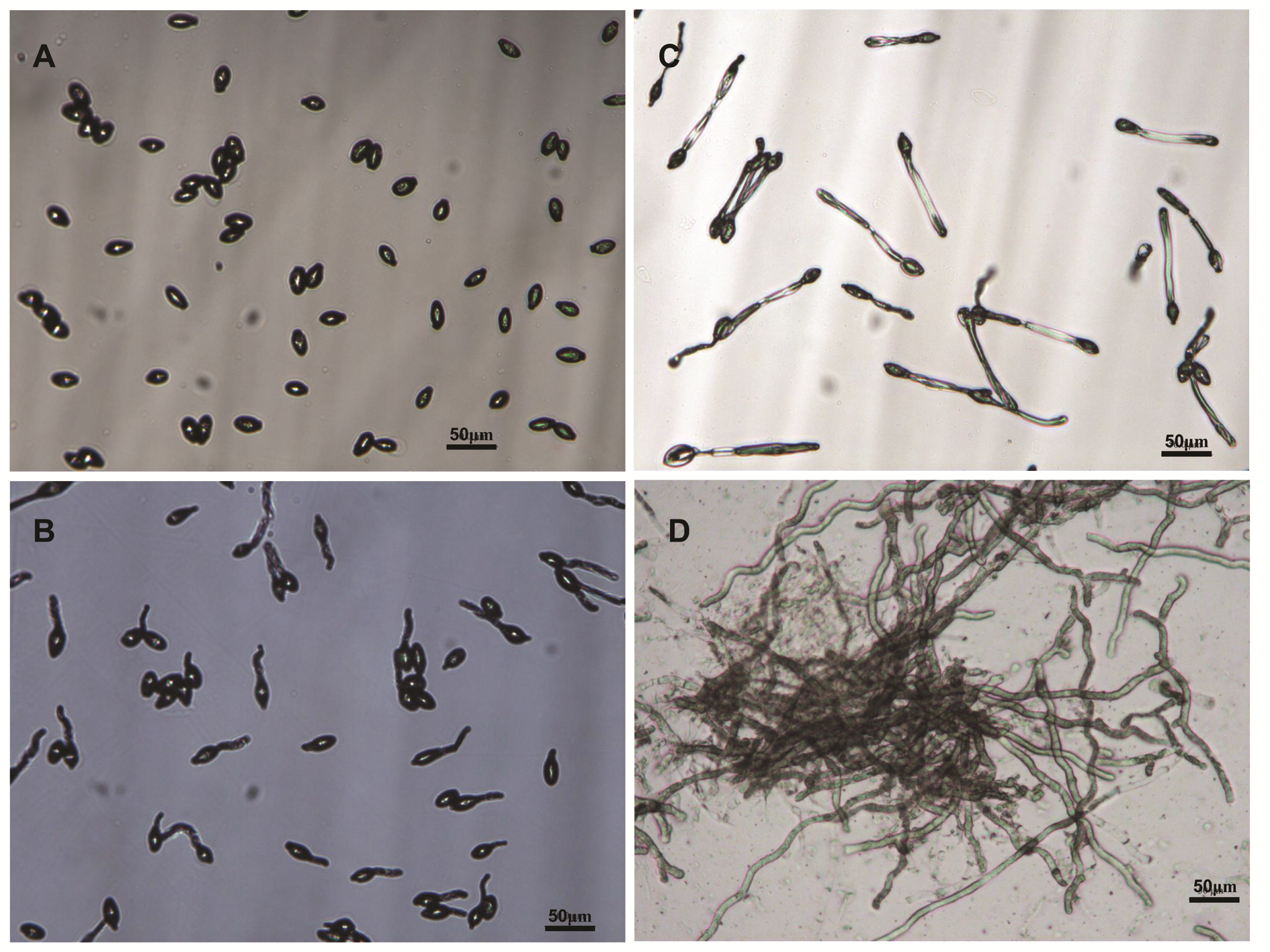

Supplement: S1 Fig — (DOCX) [file pone.0179930.s002.docx]
