# Supplementary material for: Identification and validation of reference genes for qRT-PCR studies of the obligate aphid pathogenic fungus Pandora neoaphidis during different developmental stages
Source: PLoS One. 2017 Jul 3;12(7):e0179930. doi: 10.1371/journal.pone.0179930 (PMC5495205; doi:10.1371/journal.pone.0179930)

**Supporting Information**

**S2 Fig.** Confirmation of primer specificity and amplicon length for thirteen candidate reference genes. (A) Melting curve analysis of the thirteen reference genes. (B) amplified fragments of the cDNA template of different developmental stages in 1% agarose gel electrophoresis.

**A**


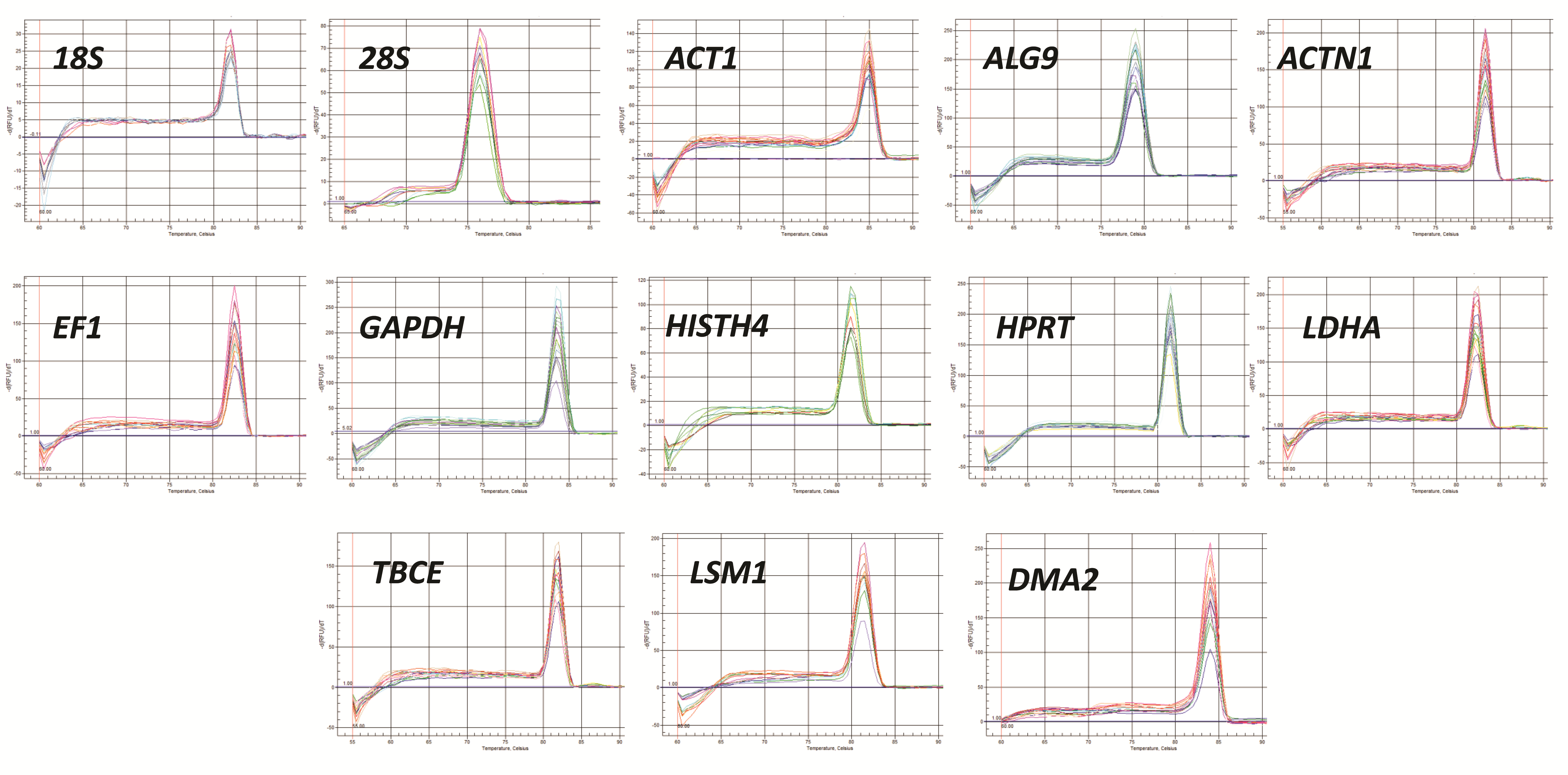


**B**


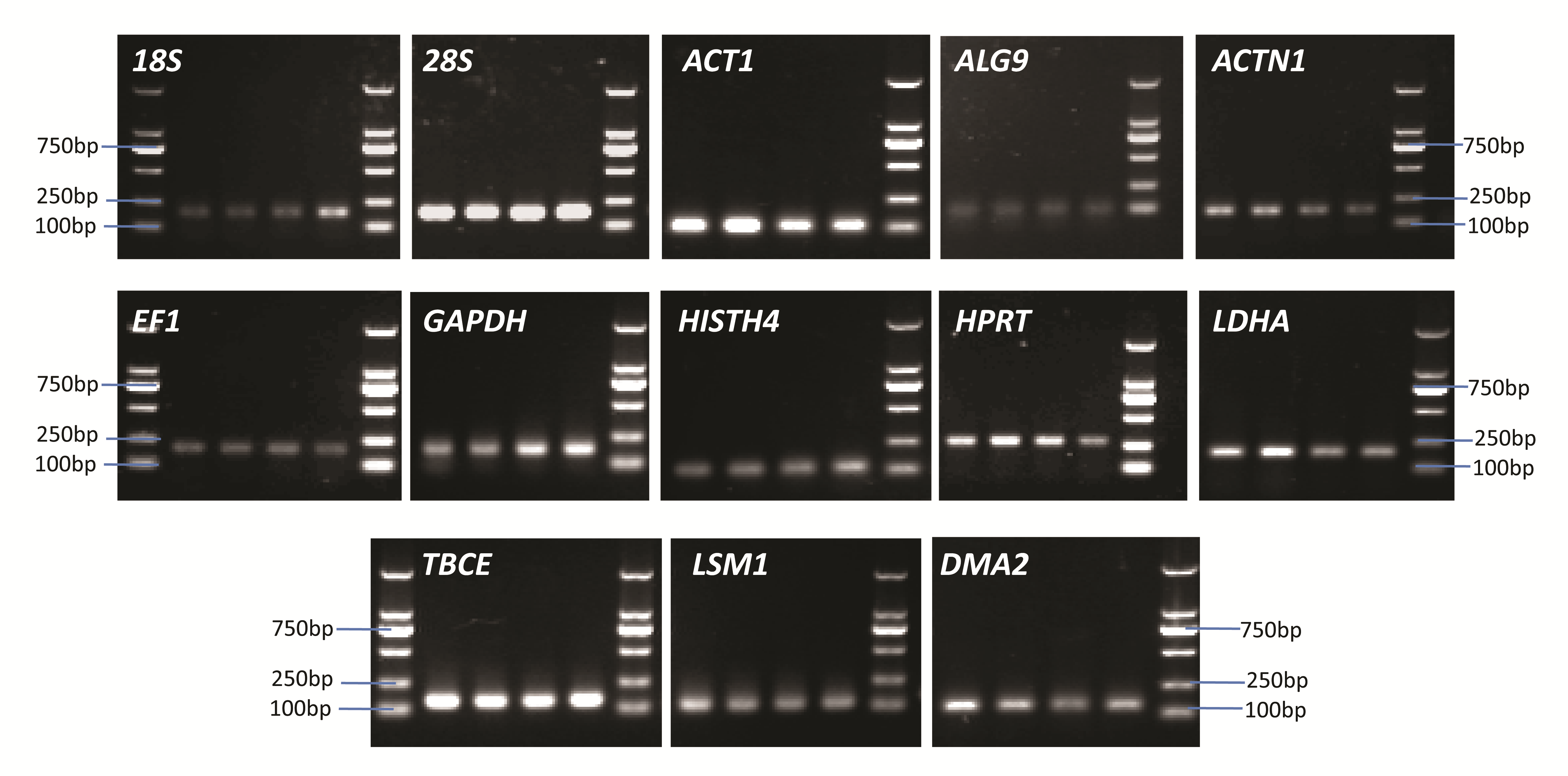

Supplement: S2 Fig — (DOCX) [file pone.0179930.s003.docx]
